# The Toxin of VapBC-1 Toxin-Antitoxin Module from *Leptospira interrogans* Is a Ribonuclease That Does Not Arrest Bacterial Growth but Affects Cell Viability

**DOI:** 10.3390/microorganisms12081660

**Published:** 2024-08-13

**Authors:** Deborah K. Damiano, Bruna O. P. Azevedo, George S. C. Fernandes, Aline F. Teixeira, Viviane M. Gonçalves, Ana L. T. O. Nascimento, Alexandre P. Y. Lopes

**Affiliations:** 1 Laboratório de Desenvolvimento de Vacinas, Instituto Butantan, Avenida Vital Brasil, 1500, São Paulo 05503-900, Brazil; deborah.damiano@usp.br (D.K.D.); brunapigatto@usp.br (B.O.P.A.); george.fernandes@usp.br (G.S.C.F.); aline.rteixeira@fundacaobutantan.org.br (A.F.T.); viviane.goncalves@butantan.gov.br (V.M.G.); ana.nascimento@butantan.gov.br (A.L.T.O.N.); 2 Programa de Pós-Graduação Interunidades em Biotecnologia, Instituto de Ciências Biomédicas, Universidade de São Paulo, Avenida Prof. Lineu Prestes, 1730, São Paulo 05508-900, Brazil

**Keywords:** toxin-antitoxin, VapBC, VapC, *Leptospira*

## Abstract

Bacterial ubiquitous Toxin-Antitoxin (TA) systems are considered to be important survival mechanisms during stress conditions. In regular environmental conditions, the antitoxin blocks the toxin, whereas during imbalanced conditions, the antitoxin concentration decreases, exposing the bacteria cell to a range of toxic events. The most evident consequence of this disequilibrium is cell growth arrest, which is the reason why TAs are generally described as active in the function of bacterial growth kinetics. Virulence-associated proteins B and C (VapBC) are a family of type II TA system, in which VapC is predicted to display the toxic ribonuclease activity while VapB counteracts this activity. Previously, using in silico data, we designated four VapBC TA modules in *Leptospira interrogans* serovar Copenhageni, the main etiological agent of human leptospirosis in Brazil. The present study aimed to obtain the proteins and functionally characterize the VapBC-1 module. The expression of the toxin gene *vapC* in *E. coli* did not decrease the cell growth rate in broth culture, as was expected to happen within active TA modules. However, interestingly, when the expression of the toxin was compared to that of the complexed toxin and antitoxin, cell viability was strongly affected, with a decrease of three orders of magnitude in colony forming unity (CFU). The assumption of the affinity between the toxin and the antitoxin was confirmed in vivo through the observation of their co-purification from cultivation of *E. coli* co-expressing *vapB-vapC* genes. RNAse activity assays showed that VapC-1 cleaves MS2 RNA and ribosomal RNA from *L. interrogans*. Our results indicate that the VapBC-1 module is a potentially functional TA system acting on targets that involve specific functions. It is very important to emphasize that the common attribution of the functionality of TA modules cannot be defined based merely on their ability to inhibit bacterial growth in a liquid medium.

## 1. Introduction

Leptospirosis is a widespread zoonosis caused by pathogenic bacteria of the genus *Leptospira* [1], an aerobic, tightly coiled spirochete [2]. *L. interrogans* is considered to be responsible for most of the cases of leptospirosis in humans [2], which are accidental and terminal hosts. Infection in humans occurs mainly via the contact of exposed tissues with the bacteria present in rat urine, and can lead to the development, in the most serious cases, to Weil’s syndrome, which causes devastation to the kidneys and other organs, possibly leading to death [1,3]. The elucidation of the first leptospiral genomes sequencing, *L. interrogans* serovar Lai [2] and *L. interrogans* serovar Copenhageni [4], were of crucial importance for understanding the molecular mechanisms of leptospirosis physiology and its pathogenicity. Currently, due to the huge improvement in DNA sequencing technologies, over 60 genomes have been elucidated and the species of the genus *Leptospira* were divided in four subclades: P1, P2, S1 and S2, which correspond, respectively, to the pathogenic, intermediate and saprophytic groups [5]. All this development was accompanied by many studies addressing genome-identified proteins that could help solve the problem of the lack of an effective vaccine through reverse vaccinology [6,7,8,9]. Furthermore, bacterial proteins related to mechanisms as secretion systems and TA systems were also addressed [10,11,12,13].

TA systems function as an adaptation mechanism and are ubiquitously present in bacteria, contributing to survival during environmental stress conditions through a dormancy strategy, acting as transcriptional regulators or modulators of cellular mRNA stability [12,14,15]. The first TA *loci* (*ccdAB*) was discovered in 1983 and initially described as being responsible for a stable maintenance mechanism of mini-F plasmid from *E. coli* in a process called post-segregational killing [16], though the term toxin-antitoxin was first used, to our knowledge, by Sobecky et al. (1996) [17]. In this study, the authors reported a fragment named *par*CBA/DE that had a role in the stable maintenance of plasmids, in which *par*DE was described as a toxin-antitoxin system that functions to stabilize plasmids by cell killing. Since then, many TA systems have been discovered in bacterial genomes and, nowadays, they are considered ubiquitous in bacteria. TA systems have been described as having possible roles in different cellular processes, including biofilm formation [18], SOS response to DNA damage [19], persistent cell formation in response to antibiotic treatment [20,21], stringent responses to amino acid starvation [22] and, in the adaptation of bacteria and archaea, to environmental stress conditions [23]. Under certain conditions, the activation of the TA system would lead cells to a reversible state of dormancy, in which they would be able to return to their regular growing state when conditions are back to normal [24]. TA genes are commonly organized in operons, coding for a toxin, which affects essential cellular processes in the host cell, and an antitoxin that forms a stable complex with the toxin, blocking its toxic effects [23,25].

Depending on the toxin and antitoxin chemical natures and how they interact, eight types of TA systems have been described [20,26,27,28]. In TA type II, both the toxin and antitoxin are proteins, and the antitoxin blocks the toxin by protein–protein interaction, neutralizing its toxic effects on the host cell. The proteins are codified by small genes that overlap or are separated by a few base pairs and are translated through a mechanism called translational coupling, implying that the efficient translation of the toxin gene depends on the translation of the preceding antitoxin gene in the bicistronic TA operon [23,29]. Under stress conditions, the labile antitoxin is degraded by proteases, altering the ratio of T:A, which, in turn, releases the toxin in the cell causing growth arrest or cell death [30,31].

Type II TA modules are divided into families, with VapBC (virulence-associated proteins B and C) being the most abundant [31,32,33]. Toxins in this family present a PIN (PilT N-terminal) domain that forms a well-defined and conserved structure among the toxins, and to which is conferred endoribonuclease activity [33]. The PIN domain is described as having an α/β/α secondary structure, with five β-sheets in the center surrounded by α-helices that, when correctly folded, assemble three to four acidic residues and a serine or threonine residue to form the catalytic site, responsible for coordinating Mg^2+^ or Mn^2+^ ions to hydrolyze RNA [33,34]. Individual VapCs cleave specific tRNAs [32,35], mRNAs [36,37] or rRNAs [38]. In contrast, it has been suggested that some PIN domain-containing proteins may bind to RNA but not act as a ribonuclease, despite the PIN domain being structurally similar to nucleases [39].

The first toxin-antitoxin module reported in a *Leptospira* spp. was a member of the ChpKI type II family published in 2001 [40]. TA *loci* of different TA families were identified in *Leptospira* spp., with nine modules, experimentally described or hypothetical, being encoded in the genome of *L. interrogans* serovar Copenhageni strain Fiocruz L1-130, including four VapBC modules, one MazEF (ChpIK) and four modules that are yet to be classified into families, according to the TADB (toxin-antitoxin database) [41,42]. To date, only VapBC-3 has been characterized [32]. In *L. interrogans* serovar Copenhageni, all nine of the TA *loci* predicted by TADB are encoded in chromosome I [4,41,42].

Recently, our group published an in silico study in which four *vapBC loci* were identified in the genome of *L. interrogans* serovar Copenhageni strain Fiocruz L1-130 and were numbered according to genome organization [42]. Among these modules, only one had been characterized [32], which was numbered in the in silico study as the VapBC-3 module, and therefore, the other three were considered putative TAs remaining to be investigated. In this work, the VapBC-1 module was characterized. We functionally describe the VapC-1 toxin as a ribonuclease, which does not inhibit the growth of *E. coli* in liquid media as normally happens with TA modules, but strongly affects cell viability with an apparent influence on oxygen availability. Ultimately, our evidence suggests that VapBC-1 is a functional TA module.

## 2. Materials and Methods

### 2.1. Bacterial Strains and Culture Conditions

*E. coli* DH5α and *E. coli* BL21 (DE3) (ThermoFisher Scientific, Waltham, MA, USA) were used for gene cloning and expression, respectively. *E. coli* clones were grown in LB medium with 20 µg·mL^−1^ of kanamycin (LB+Kan). *L. interrogans* serovar Copenhageni strain Fiocruz L1-130 was grown in EMJH medium (Difco, Franklin Lakes, NJ, USA) with 10% *v*/*v Leptospira* Enrichment EMJH (Difco, Franklin Lakes, NJ, USA) and 1% *v*/*v* rabbit serum at 30 °C for approximately 7 days for RNA extraction.

### 2.2. Cloning and Expression of vapB-1, vapC-1 and vapBC-1

The genomic sequences of *vapB-1* (locus tag LIC10867; gene ID 2772100), *vapC-1* (locus tag LIC10866; Gene ID 2770765) and the complex *vapBC-1* from *L. interrogans* serovar Copenhageni strain Fiocruz L1-130 were obtained from TADB (URL: https://bioinfo-mml.sjtu.edu.cn/TADB2/index.php, accessed on 28 January 2019). The genes were amplified individually and in tandem by PCR from genomic DNA of *Leptospira interrogans* serovar Copenhageni strain Fiocruz L1-130 using the primers listed below (Table 1).

The PCR products were cloned into plasmid pET28a (Sigma-Aldrich, St. Louis, MO, USA). Samples were analyzed in 1% agarose gel and constructions were confirmed by DNA sequencing. Clones of *E. coli* BL21(DE3) cells transformed with pET28a*-vapB-1*, pET28a*-vapC-1* and pET28a*-vapBC-1* were cultured in an LB+Kan medium until OD_600_ reached 0.6. Expression was induced with 1 mM IPTG. Cultures were kept at 37 °C for 3 h for VapB-1 and VapBC-1, and at 20 °C overnight for VapC-1. Cultures were centrifuged for 30 min at 12,000× *g*, pellet was resuspended in 50 mM Tris, 150 mM NaCl pH 8.0 and cells were lysed by sonication or high-pressure homogenizer. Soluble and insoluble fractions were separated by centrifugation for 30 min at 12,000× *g* at 4 °C. The samples were analyzed by SDS-PAGE and Western blotting.

### 2.3. Purification of VapB-1 and VapC-1

Recombinant proteins were purified via immobilized metal affinity chromatography (IMAC). VapB-1 was purified from the soluble fraction of *E. coli* extract; the sample was applied to a 1 mL Ni^+2^-Sepharose Histrap column (GE Healthcare, Chicago, IL, USA) equilibrated with 50 mM Tris, 500 mM NaCl and 20 mM Imidazole pH 8.0, and eluted with 50 mM Tris, 150 mM NaCl and 500 mM Imidazole pH 8.0. VapC-1 was purified from the insoluble fraction of *E. coli* extracts. One sample was applied to a 1 mL Ni^+2^-Sepharose Histrap HP column (GE Healthcare, Chicago, IL, USA) equilibrated with 50 mM Tris, 500 mM NaCl, 20 mM Imidazole and 8 M urea pH 8.0, refolded in the column by gradient for urea removal, and eluted with 50 mM Tris, 150 mM NaCl and 1 M Imidazole pH 8.0. The samples were analyzed by SDS-PAGE. VapB-1 was dialyzed against 50 mM Tris pH 8.0, while VapC-1 was dialyzed in steps to phase out imidazole from the initial 1 M to 500 mM, 250 mM and finally zero, in order to reduce protein precipitation and improve stability. Total protein concentration was determined by a BCA protein assay kit (Merck KGaA, Darmstadt, Germany).

### 2.4. In Vivo Pull-Down Assay

A pull-down assay was carried out to test the interaction between the co-expressed recombinant proteins VapB-1 and VapC-1. The soluble fraction of the induced *E. coli* strain BL21 (DE3), transformed with pET28a*-vapBC-1*, was applied to a Ni^+2^-Sepharose Histrap column equilibrated as described for VapB-1 purification, and eluted with 50 mM Tris, 150 mM NaCl and 1 M Imidazole pH 8.0. Samples were analyzed by SDS-PAGE.

### 2.5. Growth Kinetics and Cell Viability Assays

Positive clones from LB+Kan plates of *E. coli* BL21 (DE3) transformed with pET28a*-vapB-1*, pET28a*-vapC-1* and pET28a*-vapBC-1* were cultured in 30 mL of LB+Kan liquid medium. Cultures were kept at 37 °C under an agitation rate of 200 rpm. When the cultures reached an OD_600_ of 0.1, expression was induced with 0.1 mM IPTG. For the growth kinetics assay, OD_600_ was measured each hour and the results were plotted.

In order of test cell viability, clones of *E. coli* were grown and induced as described above, and samples were taken before and 2 h after induction. The samples were normalized to an OD_600_ of 1, serial diluted and 100 µL of 10^−6^ dilution was seeded on an LB+Kan agar. The plates were kept at 37 °C overnight in aerobic and in microaerophilic conditions, and for 72 h in an anaerobic condition. After this time, the colonies were manually counted.

### 2.6. Ribonuclease Activity Assay

The ribonuclease activity of VapC-1 was tested using MS2 RNA (Roche, Basel, Switzerland), *E. coli* tRNA (Sigma-Aldrich, St. Louis, MO, USA) and RNA extracted from *L. interrogans* serovar Copenhageni strain Fiocruz L1-130 as substrates. Total RNA was extracted by phenol–chloroform; purity and concentration were measured by spectrophotometry. Purified VapC-1 was incubated with 1 µg of RNA in a reaction buffer with 10 mM MgCl_2_ in 15 µL volume. Reactions were carried out at 37 °C for 2 h and stopped with buffer containing formamide. The effect of EDTA (Sigma-Aldrich, St. Louis, MO, USA) was tested. The results were analyzed by TBE-Urea 10% gel electrophoresis, stained with SYBR Safe DNA Gel Stain (ThermoFisher Scientific, Waltham, MA, USA).

### 2.7. Predictions of the Secondary and Tertiary (3D) Structures

The alignment of VapC-1 from *L. interrogans* and VapC from *L. kirschneri* and *L. alexanderi* was performed with the Global Align and COBALT from the BLAST tool of NCBI (URL: https://blast.ncbi.nlm.nih.gov/Blast.cgi, accessed on 18 July 2024) [43]. *L. kirschneri* PIN-domain protein (VapC) was used as a template by the automated protein structure homology-modelling server SWISS-MODEL (URL: https://swissmodel.expasy.org/, accessed on 18 July 2024) [44]. A secondary structure prediction was performed with PSIPRED (URL: http://bioinf.cs.ucl.ac.uk/psipred/, accessed on 18 July 2024) [45] and a 3D structure prediction was performed with I-TASSER (URL: https://zhanggroup.org/I-TASSER/, accessed on 18 July 2024) [46] and SwissPDB Viewer (DeepView) (URL: https://spdbv.unil.ch/, accessed on 18 July 2024) [47].

## 3. Results

### 3.1. The VapBC-1 Module from Leptospira interrogans Serovar Copenhageni

**Figure 1:**
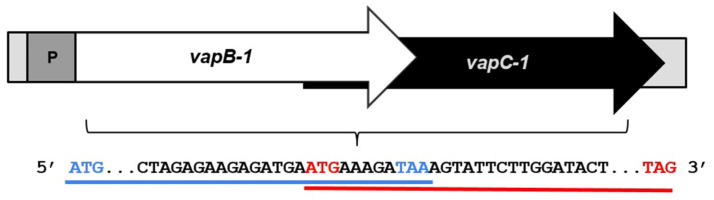
Schematic representation of leptospiral *vapBC-1* operon organization. The blue and red lines underline the partial sequence of *vapB-1* and *vapC-1*, respectively, indicating initial and stop codons. The two genes overlap by 11 nucleotides. P indicates the promoter.

The leptospiral *vapBC-1* module consists of two genes, organized in a bicistronic operon: the antitoxin *vapB-1* is a sequence of 219 base pairs, encoding a protein of 72 amino acids, while the toxin *vapC-1* is a sequence of 444 base pairs, encoding a protein of 147 amino acids. *vapB-1* and *vapC-1* have 11 nucleotides in common, meaning that the start codon of *vapc-1* overlaps with the end of the *vapB-1* sequence. In this type of genetic arrangement, where genes are separated or overlapped by a few nucleotides, a phenomenon called translational coupling occurs, resulting in translation interdependence (Figure 1).

**Figure 2:**
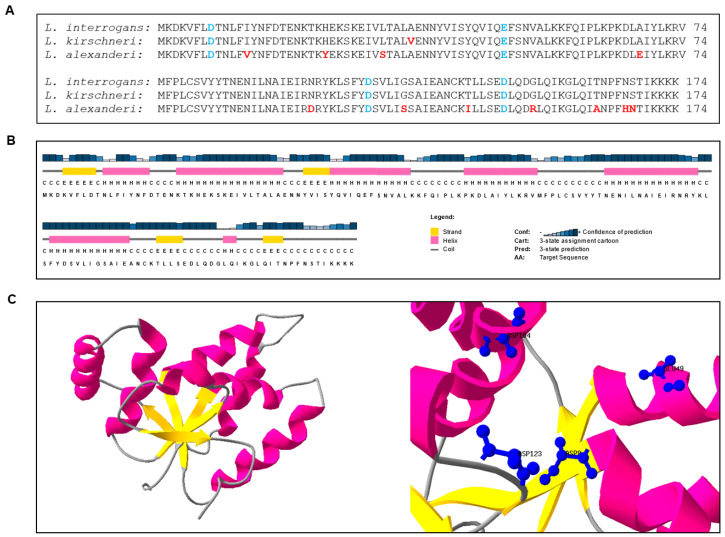
In silico analysis of VapC-1 from *L. interrogans*. (**A**) Alignment of the sequences of VapC-1 from *L. interrogans* and the PIN domain containing proteins from *L. kirschneri* and *L. alexanderi*. Highlighted amino acids in blue consist of the four acidic residues essential for the catalytic activity of the PIN domain. Amino acid mismatches are highlighted in red. Alignment was conducted with the COBALT tool. (**B**) Prediction of the VapC-1 secondary structure by PSIPRED using the primary sequence of the protein. (**C**) Tertiary (3D) structure of VapC-1 modelled with SwissPDB Viewer (DeepView). The left panel shows the structure model (ribbon) and the right panel shows the four conserved acidic residues responsible for coordinating metal ions in the catalytic site.

VapC-1 from *L. interrogans* sorovar Copenhageni primary sequence presents the four conserved acidic residues required to form the PIN domain (Figure 2A), to which is attributed the ribonuclease activity [33]. The alignment of primary sequences (Figure 2A) showed that VapC-1 is very highly conserved among two other species of pathogenic *Leptospira*: *L. kirschneri* (99%) and *L. alexanderi* (93%), contrasting with the low identity found among the ones from *L. interrogans* sorovar Copenhageni that vary from 18 to 23% [42]. The prediction of the secondary structure shows that VapC-1 contains four regions consistent with β-sheet structures and seven regions consistent with α-helixes (Figure 2B). Considering that the 3D structure of an enzyme correlates to its biochemical activity, we have submitted the primary sequence of VapC-1 to I-TASSER for 3D modelling using the AlphaFold DB model (AOA828Y4G1) from *L. kirschneri* as a template. The predicted 3D structure shows that β-sheets are present in the center of the structure, surrounded by α-helixes, as happens in experimentally solved X-ray structures. Additionally, the conserved residues responsible for coordinating metal ions in the catalytic site are shown in detail (Figure 2C). All features are consistent with the description of the PIN domain, meaning that VapC-1 has the necessary features to be an active ribonuclease.

### 3.2. Production of Recombinant VapB-1 and VapC-1

VapB-1 and VapC-1 recombinant proteins were produced in *E. coli* BL21 (DE3). VapB-1 antitoxin was produced in the soluble fraction of *E. coli* extract, while VapC-1 toxin was produced in the insoluble fraction. Proteins were purified via immobilized metal affinity chromatography (Figure S1) and dialyzed for imidazole removal as described in the Materials and Methods Section. It is important to note that for VapC-1, it was necessary to solubilize the protein using 8 M urea, refold it in the column by gradient removal of urea and dialyze in steps to phase out imidazole in order to reduce protein precipitation and improve stability. Recombinant proteins were then quantified and stored at −20 °C for use in future experiments.

### 3.3. The Toxin VapC-1 and Antitoxin VapB-1 Interact In Vivo but Not In Vitro

**Figure 3:**
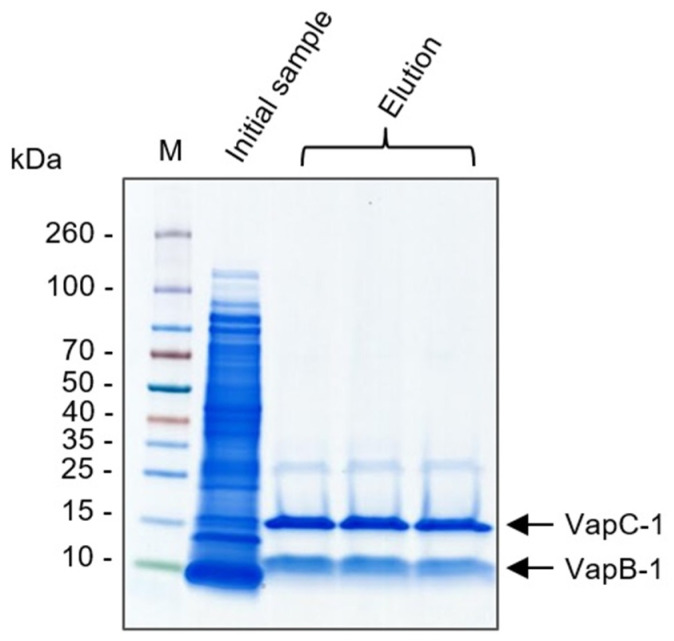
Analysis of the pull-down assay by SDS-PAGE. The soluble fraction of VapBC-1 was applied to a Ni^+2^ column, VapC-1 adsorbs to the column through C-terminal 6xHis tag and VapB-1 binds to VapC-1. Proteins are co-purified, suggesting VapB-1 and VapC-1 interact in vivo. Initial sample of soluble VapBC-1 and elution fractions are shown. M = molecular marker (kDa). Arrows indicate VapB-1 and VapC-1 bands.

Both *vapB* and *vapC* genes were cloned in tandem into the pET28a vector as they appear in the *vapBC-1* operon, the 6xhistidine tag was fused only at C-terminus of VapC-1 and the bicistronic mRNA was expressed as two independent proteins, as expected. In order to verify whether the antitoxin VapB-1 and its cognate antitoxin VapC-1 interact in vivo, a pull-down assay was performed through the use of an IMAC chromatography by applying the soluble fraction of lysed *E. coli* total extract. The elution fractions obtained from the chromatography were analyzed by SDS-PAGE. Figure 3 shows that VapB-1 and VapC-1 were co-purified, demonstrating that the nontagged VapB-1 was able to interact with VapC-1. As a result, both proteins were pulled down from the chromatography column, indicating the in vivo affinity between the proteins in *E. coli*.

In addition, in order to evaluate whether recombinant VapB-1 and VapC-1 purified separately interact in vitro, a dot blot assay was performed. VapC-1 was adsorbed on a membrane exposed to a solution containing VapB-1. Primary antibodies, obtained from mice sera, were used to detect VapB-1 blotted in the membrane. The results showed that VapB-1 was not detected by anti-VapB antibodies, indicating that purified VapB-1 did not interact with purified, refolded VapC-1 (Figure S2).

### 3.4. vapC-1 Expression in E. coli Significantly Affects Cell Viability, but Does Not Arrest Growth in Liquid Medium Culture

**Figure 4:**
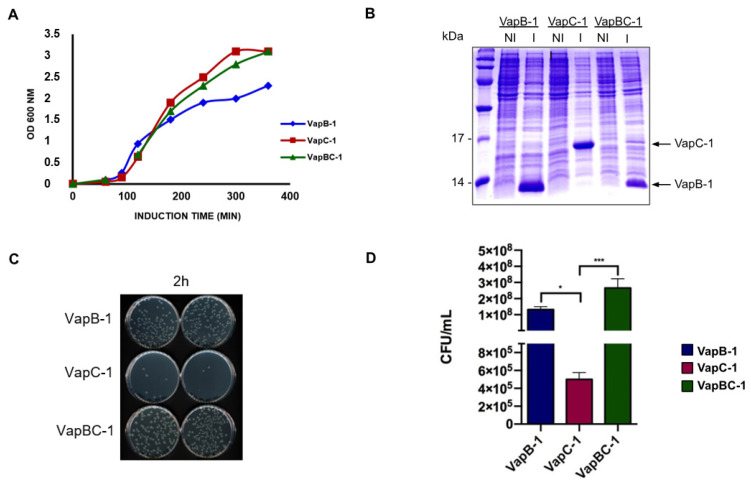
Evaluation of VapC-1 influence on *E. coli* growth and cell viability. (**A**) Growth kinetics in *E. coli* BL21 (DE3). *E. coli* pET28a-*vapB-1*, pET28a-*vapC-1* and pET28a-*vapBC-1* were induced at OD_600_ = 0.1 and optical density was measured for 5 h. (**B**) Analysis of protein production during growth kinetics assay in *E. coli* BL21 (DE3). Samples were taken pre-induction and 5 h post-induction. (**C**) Viability assay plates 2 h post-induction. Induced *E. coli* pET28a-*vapB-1*, pET28a-*vapC-1* and pET28a-*vapBC-1* were plated in LB+Kan agar 2 h after induction. (**D**) Viability assay. CFU were counted and values were plotted. *vapC-1* expression inhibits formation of colonies on solid medium. Results are an average of four independent experiments and show that *vapC-1* expression affects CFU but not cell growth on liquid medium. One-way ANOVA was used for statistical analysis. * *p* < 0.05, *** *p* < 0.001.

*E. coli* transformed with pET28a-*vapB-1,* pET28a-*vapC-1* and pET28a-*vapBC-1* were cultured on LB+Kan liquid medium and induced with 0.1 mM IPTG (OD_600_ of 0.1). After induction, the optical density was followed each hour for five hours to evaluate the influence of the produced proteins in bacterial growth kinetics. Complementarily, aiming to further analyze any possible effect on colony formation, the cultures were serially diluted and plated on LB+Kan agar. Two hours after induction, samples were plated for CFU counting. We observed that the expression of the toxin did not cause a decrease in the culture’s optical density in relation to the toxin-antitoxin complex and that all constructions were confirmed to synthetize the coding proteins (Figure 4A,B). However, cell viability studies via CFU count showed a decrease of three orders of magnitude in viable cells 2 h after expression, compared to complexed toxin-antitoxin and antitoxin (Figure 4C,D). Careful observation of Figure 4A,D shows us that apparently the synthesis of VapB-1 in both liquid and solid media has a small effect on *E. coli* growth that should not be ruled out, but for which we have no hypothesis other than some intrinsic toxicity of this protein on *E. coli*. Notwithstanding, this effect cannot be attributed to the role of this TA module on the homologous bacteria *L. interrogans*.

**Figure 5:**
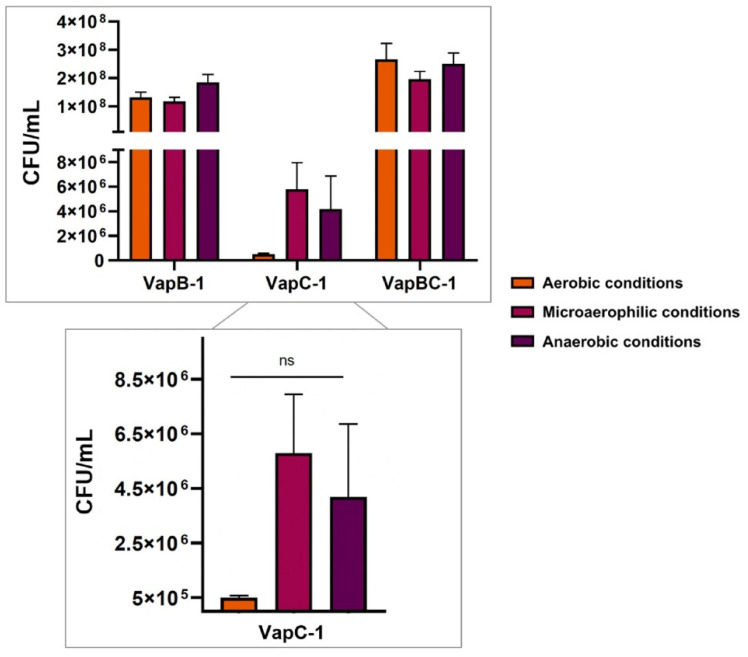
*E. coli* viability assay performed in aerobic, microaerophilic and anaerobic conditions. CFU were counted and results were plotted. Induced *E. coli* pET28a-*vapB-1*, pET28a-*vapC-1* and pET28a-*vapBC-1* were plated in LB+Kan agar 2 h after induction. *E. coli* pET28a-*vapC-1* produced an increased number of colonies under low oxygen availability when compared to aerobic conditions. One-way ANOVA was used for statistical analysis, with *p*-value < 0.05. ns = not significant.

In order to find an explanation to the decrease in colony number in solid medium, as opposed to the liquid culture, which kept growing, we explored whether the availability of oxygen had any influence in growth, as one of the possible differences between liquid and solid media is oxygen distribution. We observed that *E. coli* producing VapC-1 did indeed form more colonies in solid media when grown in microaerophilic and anaerobic conditions compared to aerobic conditions (Figure 5). Thus, our results indicate that the availability of oxygen may be a factor interfering in the colony forming of bacteria producing VapC-1, indicating that some mRNA coding protein exclusive of the aerobic metabolism pathway could potentially be one of VapC-1 targets. However, as CFU counting was not comparable to *E. coli* producing VapB-1 and VapBC-1 (Figure 5), we cannot exclude other mRNA targets from a distinct metabolic pathway affecting this phenomenon.

### 3.5. VapC-1 Cleaves MS2 RNA and Total RNA from L. interrogans Serovar Copenhageni Fiocruz L1-130

**Figure 6:**
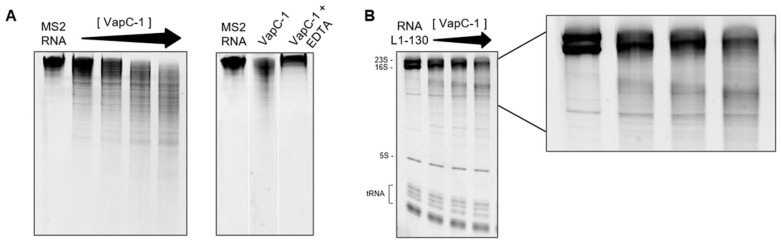
Analysis of ribonuclease activity in 10% TBE-Urea gel. VapC-1 was incubated with RNA at 37 °C for 2 h in the presence of 10 mM MgCl_2_. Gels were stained with SYBR Safe DNA Gel Stain. (**A**) A total of 1 µg of MS2 RNA substrate incubated with 0.25, 0.5, 1 and 2 µg of VapC-1. The effect of 100 mM EDTA was tested. (**B**) A total of 1 µg of RNA from *L. interrogans* serovar Copenhageni str. Fiocruz L1-130 substrate incubated with 0.5, 1 and 2 µg of VapC-1. VapC-1 degrades MS2 RNA, subunits 23S and 16S of total leptospiral RNA and its activity is dependent of MgCl_2_.

To investigate whether VapC-1 from *L. interrogans* serovar Copenhageni really acts as a ribonuclease, we tested the activity of the VapC-1 module using *E. coli* tRNA, MS2 RNA and total RNA from *L. interrogans* serovar Copenhageni as substrates. RNAse activity in vitro assays showed that recombinant VapC-1 does not cleave *E. coli* tRNA (Figure S3), but cleaves MS2 RNA (Figure 6A) and total RNA, hydrolyzing 23S and 16S ribosomal subunits but not the 5S subunit and tRNAs, which indicates that it is not an unspecific ribonuclease (Figure 6B). Differently from what was observed in vivo, recombinant VapB-1 did not inhibit VapC-1 activity (Figure S4). Nonetheless, this result agrees with the lack of interaction seen in the dot blot assay performed to evaluate the in vitro interaction. Moreover, Figure 6 shows that VapC-1 acts as a ribonuclease in a dose-dependent manner and that the addition of EDTA abolished RNA cleavage, indicating that it is a divalent metal-dependent enzyme like other VapC toxins (only MgCl_2_ was tested).

The in vitro affinity between the recombinant toxin and antitoxin was tested by the dot blot assay, which showed that VapB-1 is not able to interact with VapC-1, likely due to some imperfect folding during protein synthesis. This lack of interaction between the toxin and the cognate antitoxin may support the fact that VapB-1 was not able to effectively inhibit VapC-1 ribonuclease activity in vitro.

## 4. Discussion

Since the discovery of the first toxin-antitoxin module in 1983 until now, thousands of articles reporting the high diversity of TA systems, including types, families, unique module structures and the variation of numbers within a single bacterium, have been published. Despite all these efforts, the biological functions of TAs are still controversial and undefined. Hence, the importance of continuing investigations to identify and characterize new TAs.

As previously reported by our group, *L. interrogans* serovar Copenhageni possesses four members of type II VapBC family, and to date, only VapBC-3 had been characterized. Here we describe VapBC-1 as a new active TA module of *Leptospira*. Our in silico studies showed that the VapC-1 toxin presents the four acidic residues essential for the ribonuclease properties of the PIN domain, and according to secondary and tertiary structure predictions, it has structures consistent with four β-sheets and seven α-helixes. These three features are required for the formation of the PIN domain [34], indicating VapC-1 has all the key features to potentially display this enzymatic activity. Consistently, VapC-1 displayed ribonuclease activity towards *L. interrogans* total RNA, acting selectively against some RNA forms, specifically the 23S and 16S subunits, but not 5S and tRNAs.

When functioning typically, the toxin and antitoxin must be able to physically interact to keep the equilibrium of the complex, maintaining the toxin inhibited in non-stressing conditions. Our initial experiments confirmed the heterologous co-expression of *vapB-1* and *vapC-1* cloned in tandem, exactly like they are found in the *Leptospira* operon, which resulted in the production of VapB-1 and VapC-1. As indicated by the pull-down assay, the toxin and its cognate antitoxin physically interact with each other when produced in vivo in *E. coli*, as is observed in many other reports from different type II families, including toxin-antitoxin VapBC systems from *L. interrogans* sorovar Copenhageni [32,48,49,50,51]. In contrast with what is seen in the dot blot, recombinant VapB-1 was not able to interact with recombinant refolded VapC-1, which could explain the enzymatic results, where, despite the fact that VapC-1 exhibited ribonuclease activity, the presence of VapB-1 could not inhibit RNA cleavage. Ultimately, the results indicate that when VapB-1 and VapC-1 were synthesized in sequence in their native conformation, they were able to interact with each other; however, when they were produced separately, needing in vitro processing, they lost the ability to interact, likely due to minor errors during the folding of the proteins.

As commonly found in the TA systems literature, the first step in characterizing a new TA module begins by screening whether the expression of the toxin gene would cause the growth arrest of a heterologous bacterium and only then can it be confirmed that it is an actual bona fide element, as described by Gupta (2009) [52]. Our results showed that, despite not having an effect on *E. coli* growth kinetics in liquid medium, VapC-1 inhibited the formation of bacterial colonies in solid medium, indicating that it does affect the ability of cells to form new colonies. Similar behavior was described by Muthuramalingam et al. (2019) [53], who observed that the expression of the toxin ParE from the TA ParDE system from *Pseudomonas aeruginosa* has no apparent effect on the optical density of the culture in a liquid medium, but it affects the colony-forming ability in a solid medium. The results obtained indicate that the inhibition of bacterial growth by optical density measurement may not be enough to attribute function to TA systems.

In order to evaluate whether the availability of oxygen in the liquid or solid media could interfere with growth, anaerobic and microaerophilic conditions were studied. Our results lead us to hypothesize that the availability of oxygen may influence colony formation of *E. coli* expressing *vapC-1* by action on mRNA targets exclusive of the aerobic metabolism pathway impacting this phenomenon. A study by Fortuin and co. (2020) [54] comparing the transcriptome of *E. coli* cells growing in liquid or solid media showed that the presence of proteins in the suf operon that are involved in iron mobilization and swarming motility was associated exclusively with single colony profiles. Meanwhile, those proteins involved in motility such as MotA, MotB, FliH, Flip, FliD and FliJ were associated exclusively with cells grown in liquid culture. This is in agreement with the established knowledge that many important determinants associated with virulence and host cell adhesion are exclusively expressed during growth on solid media [54,55]. The achievement of this work gave us a reasonable hypothesis when considering our results that distinguish growth in liquid and on solid media. There are circumstances that could explain why bacteria may grow in liquid but not on solid media. VapC-1 could have as one of its biological targets a critical mRNA associated with the expression of genes related to iron mobilization and swarming motility, leading to the loss of the ability to form colonies. If bacterial cells are injured because of the presence of VapC-1 toxin, it could result in loss of viability by plate counts and the formation of nonculturable phenotypes.

VapC toxins are PIN-domain endonucleases that have been shown to hydrolyze different RNAs that very likely are the true biological targets, such as initiator tRNA^fMet^ [32,35] and other tRNAs [56,57,58], the sarcin–ricin loop of 23S rRNA [38] and mRNAs [36,59], ultimately causing general blockage of the translation machinery and affecting specific protein synthesis. Additionally, unspecific large substrates containing a varied range of nucleotide sequences have been used for the purpose of screening ribonuclease activity, the most used RNA from bacteriophage MS2 [59,60,61], which was used first in our experiments to test nuclease activity. Another substrate used in our experiments was the leptospiral total RNA from the homologous bacteria originating VapC-1, which has the benefit of representing a potential biological target. The activity we found against 23S and 16S subunits in vitro would lead to a complete block of translation in vivo; nevertheless, the selectivity we found in arresting bacterial growth only on solid medium, together with the clear influence of oxygen availability on CFU, seems to suggest that the VapBC-1 module is more likely to act on targets involving specific functions related to environmental conditions. Evidently, we cannot infer that these activities could be transposed from *E. coli* to *L. interrogans,* but due to all the difficulties associated with cultivating *Leptospira* spp. and similar for bacterial survival strategies and general metabolic mechanisms, it seems reasonable to suggest that this hypothesis could be considered. Further studies to discover the actual biological targets of VapC-1 and the function of VapBC-1 are needed.

Until now, two type II TA modules from *Leptospira* have been experimentally characterized: a ChpIK module from *L. interrogans* serovar Icterohaemorrhagiae strain Lai [62], VapBC of *L. interrogans* serovar Lai [63] and the homologous further characterized VapBC of *L. interrogans* serovar Copenhageni [32]. The latter refers to VapBC-3 according to genome organization [42]. In this work, we functionally characterize VapC-1 from *L. interrogans* serovar Copenhageni, and the results obtained indicate that VapBC-1 may be a functional module, raising the question of the usual attribution of functionality to TA modules based on the arrest of bacterial growth. The results presented here, in combination with the variability of TA modules described in the literature, highlights the need to identify and characterize new TA modules in order to understand their role in bacteria.

## Figures and Tables

**Table 1:** Primers used in the amplification of genes and expression vector constructs. Restriction enzyme recognition sites are underlined, histidine tag sequence is presented in bold upper-case letters, followed by coding region sequences.

Gene	Primer Sequence	Restriction Enzyme	Construction
*vapB-1*	F: 5′ agccatgggcCATCACCATCACCATCACATGA AGAATATTACGTTTAGAG 3′	*Nco* I	pET28a-*vapB-1*
	R: 5′ gagcctcgagTTATTATCTTTCATTCATCTCTTC 3′	*Xho* I	
*vapC-1*	F: 5′ agccatgggcCATCACCATCACCATCACATGA AAGATAAAGTATTCTTGG 3′	*Nco* I	pET28a-*vapC-1*
	R: 5′ gagcctcgagTTACTACTTTTTCTTTTTTATTGTGC 3′	*Xho* I	
*vapBC-1*	F: 5′ gagccatgggcATGAAGAATATTACGTTTAGAG 3′	*Nco* I	pET28a-*vapBC-1*
	R: 5′ gagcctcgagCTTTTTCTTTTTTATTGTGC 3′	*Xho* I	

## Data Availability

The original contributions presented in the study are included in the article/Supplementary Material, further inquiries can be directed to the corresponding author.

## Supplementary Materials

The following supporting information can be downloaded at https://www.mdpi.com/article/10.3390/microorganisms12081660/s1, Figure S1: Analysis of purified recombinant proteins VapB-1 and VapC-1 by SDS-PAGE. Figure S2: Dot blot analysis. Figure S3: Analysis of ribonuclease activity assay in 10% TBE-Urea gel (*E. coli* tRNA). Figure S4: Analysis of ribonuclease activity assay in 10% TBE-Urea gel (total RNA from *L. interrogans* serovar Copenhageni).
