# Supplementary material for: The Toxin of VapBC-1 Toxin-Antitoxin Module from Leptospira interrogans Is a Ribonuclease That Does Not Arrest Bacterial Growth but Affects Cell Viability
Source: Microorganisms. 2024 Aug 13;12(8):1660. doi: 10.3390/microorganisms12081660 (PMC11356721; doi:10.3390/microorganisms12081660)
Supplement: Supplementary file 1 [file microorganisms-12-01660-s001.zip › microorganisms-3140529-supplementary.pdf]

Supplementary Material

The Toxin of VapBC-1 Toxin-Antitoxin Module from *Leptospira interrogans* is a Ribonuclease that Does Not Arrest Bacterial Growth but Affects Cell Viability

Materials and Methods

Antiserum production in mice immunized with VapB-1

Female BALB/c mice were subcutaneously immunized with 5 µg of VapB-1 adsorbed in $Al(OH)_3$ (12.5% v/v per dose), with three booster doses administered at approximately 15-day intervals. The animals were bled via retro-orbital plexus before the first immunization and 15 days after each subsequent immunization. The collected blood was kept at room temperature for about 30 min, and the clot was centrifuged at 2000 g for five min. After centrifugation, the collected serum was stored at -20°C. The sera were titrated by ELISA.

Ethics statement: This work used mice for obtaining VapB anti-serum. During the experiment, animals were supplied with food and water *ad libitum* and experimental protocols were previously approved by the Ethical Committee for Animal Research of the Butantan Institute (CEUA-IB Nº 3200120118).

Dot Blot analysis

Recombinant toxin VapC-1 (2 µg) was adsorbed in triplicate on a nitrocellulose membrane (BioRad, California, USA). The membrane was dried in room temperature and blocked overnight with 10% non-fat dry milk in PBS-Tween 20, washed three times for five min with PBS-T and incubated with 3 µg/mL of recombinant VapB-1 protein in PBS 1x for 2 h. Membranes were washed again and incubated with polyclonal antibodies against VapB-1, diluted 1:1000 in 10% non-fat dry milk in PBS-T, for 1 h. After three washes, the membrane was incubated with secondary antibody, anti-mouse IgG-peroxidase (Invitrogen, Massachusetts, USA), diluted 1:1000 in 10% non-fat dry milk in PBS-T, for 1 h. Membrane was further washed and developed by chemiluminescence using SuperSignal® West Dura Extended Duration (ThermoFisher Scientific, Massachusetts, USA). Image was visualized in Amersham Imager 600 (GE Healthcare, Illinois, USA).

Results

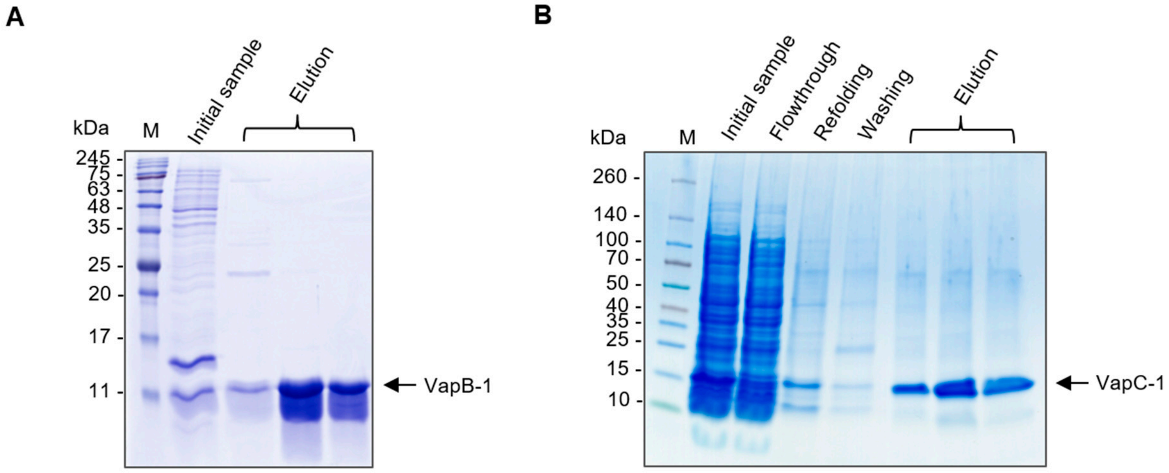

Figure S1. Analysis of purified recombinant proteins VapB-1 and VapC-1 by SDS-PAGE. Proteins were purified via IMAC using a Ni^{2+} column and eluted with imidazole in three fractions. (**a**) VapB-1 was purified from the soluble fraction of *E. coli* extracts. (**b**) VapC-1 purified from the inclusion bodies of *E. coli* extracts. M = Molecular marker (kDa). Arrows indicate VapB-1 and VapC-1 bands.

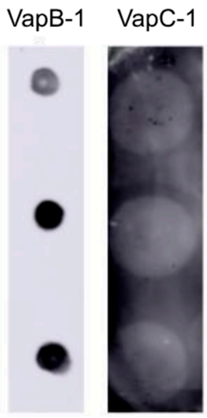

Figure S2. Dot blot analysis. Recombinant VapC-1 (2 µg) was adsorbed to a membrane (triplicate) and incubated with 3 µg/mL of VapB-1 in PBS 1x. The membrane was incubated with primary antibodies against VapB-1 from mice and with secondary antibody anti-mouse IgG-peroxidase. Recombinant VapB-1 (2µg) served as a control. Purified VapB-1 did not interact with VapC-1.

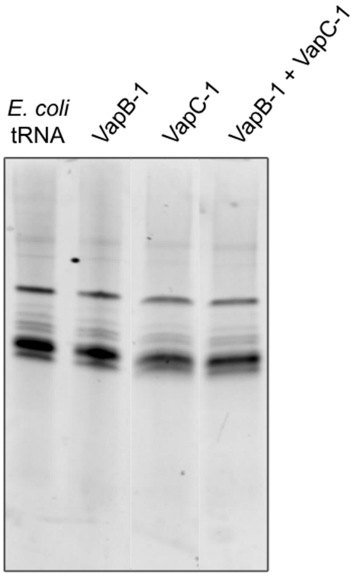

Figure S3. Analysis of ribonuclease activity towards *E. coli* tRNA in 10% TBE-Urea gel. VapB-1 (1 μg), VapC-1 (1 μg) and VapC-1 (1 μg) + VapB-1 (1 μg) were incubated with *E. coli* tRNA at 37°C for 2 h in the presence of 10 mM $MgCl_2$. Gels were stained with SYBR Safe DNA Gel Stain. VapC-1 does not hydrolyze *E. coli* tRNA.

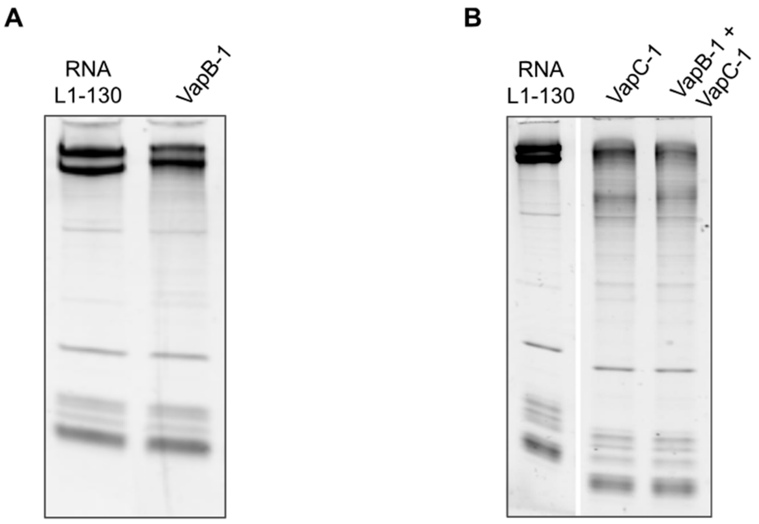

Figure S4. Analysis of ribonuclease activity assay in 10% TBE-Urea gel. Total RNA extracted from *L. interrogans* serovar Copenhageni L1-130 (1 μg) was used as substrate and reactions were carried out at 37°C for 2 hours in the presence of 10 mM $MgCl_2$. Gels were stained with SYBR Safe DNA Gel Stain. (**a**) VapB-1 (1 μg). (**b**) VapC-1 (2 μg) and VapB-1 (2 μg) pre-incubated with VapC-1 (2 μg). VapB-1 did not cleave total *L. interrogans* RNA and did not inhibit VapC-1 ribonuclease activity.
